# The Ladies’ Sanitary Association of London

**Published:** 1862-08

**Authors:** 


The Ladies' Sanitary Association of London.—This association is extending the sphere of its usefulness wider and wider. The tracts are not only widely circulated in England, but they have reached continental cities, where the health authorities have in many instances reproduced them. A London cotemporary thus speaks of the sphere to which this excellent association devotes its labors:

"It enters the mechanic's room and the poor man's cottage, and talks, in a woman's voice, to mechanics' and poor men's wives. It points out that though the court may be drained, or the cesspool removed, the living room must have its windows opened; that though the infant may have been vaccinated, it must be fed and clothed in a proper manner; that notwithstanding warm under-clothing and coals have been supplied by the parish or by public charity, soap and warm water must be provided at home. The children laid up with scarlet fever may have been seen by the doctor, but his aid will be but trifling if they are left half suffocated in the close atmosphere which reeks up from a dirty and slovenly bed. The mothers of families are taught that true economy exists not simply in buying in a cheap market, but in knowing how afterwards to employ their bargains to the best advantage. There is a good cheap cookery, as well as a bad cheap one; an effective cheap mode of dressing, as well as an inefficient cheap style of apparel. The former are shown to be easily substituted for the latter. These are, after all, things in which most reform is needed within the poor man's dwelling, and which reform, when decorated by the thrift and comfort of hygienically regenerated wives and daughters, will do more towards the winning of our working men from skittle-grounds and gin-shops than a millenium of Exeter Hall demonstrations."

